# Maladie thromboembolique veineuse dans la région de Sidi Bel Abbes, Algérie: fréquence et facteurs de risque

**DOI:** 10.11604/pamj.2013.16.45.2620

**Published:** 2013-10-10

**Authors:** Nourelhouda Chalal, Abbassia Demmouche

**Affiliations:** 1 Département de biologie, Faculté des sciences, Université Djillali Liabes, Sidi Bel Abbes, Algérie

**Keywords:** Maladie thromboembolique veineuse, facteurs de risque, fréquence, Sidi Bel Abbes, Venous thromboembolism, risk factors, frequency, Sidi Bel Abbes

## Abstract

**Introduction:**

La maladie thromboembolique veineuse (MTEV) présente par ses deux entités cliniques: thrombose veineuse profonde (TVP) et embolie pulmonaire (EP), est une pathologie fréquente ayant une forte morbi-mortalité. En Algérie, cette pathologie prend de plus en plus de l'ampleur, en l'absence de toute publication révélant sa fréquence et le pouvoir thrombogène des facteurs de risque qui lui sont corrélés. Notre étude a pour objectif de déterminer la fréquence et les facteurs de risque de ce type d'affection dans la région de Sidi Bel Abbes, Algérie.

**Méthodes:**

Il s'agit d'une étude rétrospective allant du 1^er^ janvier 2006 au 10 juin 2012 ciblant les patients hospitalisés pour TVP et /ou EP au sein du service de cardiologie du CHU de Sidi Bel Abbes.

**Résultats:**

183 patients atteints de la MTEV dont 112 femmes (61.2%) d’âge moyen 46.4 ± 17.9 et 71 hommes (38.7%) d’âge moyen 51.5 ± 17.7 ont été notés. 146 cas parmi eux (79.7%) présentaient une TVP isolée, alors que 37 autres (20.2%) étaient atteints d'EP, dont 16 cas de TVP associée. Les facteurs de risque les plus fréquents enregistrés en cas de TVP sont surtout: l'immobilité, l'hypertension, la chirurgie, et la contraception orale, tandis que: l'immobilité, la chirurgie, l'hypertension et les fractures sont les facteurs de risques les plus incriminés en cas d'EP. 24.7% des patients présentaient plusieurs facteurs de risque. L'antécédent personnel de la MTEV, était présent dans 12.02% des cas. 97.5% des TVP ont touché les membres inférieurs mais seulement 2.5% des TVP étaient localisés au niveau des membres supérieurs.

**Conclusion:**

Au terme de notre étude, et en dépit de sa fréquence non alarmante, il serait indispensable d'envisager l'adoption d'une stratégie prophylactique adéquate afin de lutter contre le développement redoutable de ce genre d'affection dans la région de Sidi Bel Abbes.

## Introduction

La maladie thromboembolique veineuse sous ses 2 aspects cliniques: thrombose veineuse profonde et embolie pulmonaire, est une affection fréquente avec une incidence annuelle de 1 à 2 cas pour 1000 personnes dans la population générale et dont le pronostic vital et fonctionnel peut s'avérer grave [1, 2]. Par son impact sur la morbi-mortalité et les coûts médicaux, la MTEV représente toujours un enjeu majeur de santé publique. Rare pendant l'enfance, avec un taux négligeable [4]. En Algérie, ce type d'affection prend de plus en plus de l'ampleur, en l'absence de publications révélant sa fréquence et le poids thrombogène des facteurs de risque qui lui sont associés.

L’étude rétrospective qu'on a menée nous aide à mettre un peu plus en lumière la réalité de la maladie thromboembolique veineuse dans la région de Sidi Bel Abbes en déterminant sa fréquence et ses facteurs de risque.

## Méthodes

Il s'agit d'une étude rétrospective, s’étalant du 1^er^ janvier 2006 au 10 juin 2012, ciblant les patients hospitalisés pour une TVP et /ou une EP, au sein du service de cardiologie du CHU de Sidi Bel Abbes. Les patients inclus dans cette étude, sont ceux dont le diagnostic s'est confirmé par: écho- Doppler vasculaire, angioscanner thoracique. Les données concernant l’âge, les facteurs de risque, et la localisation de cette pathologie, ont été retenues à la suite de l'examen des dossiers médicaux. Les graphes et le calcul de l’âge moyen ont été réalisés à l'aide du logiciel Statview version 5.

## Résultats

Durant cette période, 183 patients atteints de la MTEV se sont présentés, dont 112 femmes (61.2%) d’âge moyen 46.4 ± 17.9 et 71 hommes (38.7%) d’âge moyen 51.5 ± 17.7 (Tableau 1).

**Tableau 1:** Caractéristiques des patients atteints de la MTEV

Variables	Patients atteints (N = 183)	Homes (N = 71)	Femmes (N = 112)
**Age (ans)**	48.4 ± 17.9	51.5 ± 17.7	46.4 ±17.9
**TVP**	146(79.7)	52(73.2)	94(83.9)
**EP**	37(20.2	19(26.7)	18(16)

Valeurs exprimées en n(%), moyenne ± écart type pour l’âge

**Figure 1:**
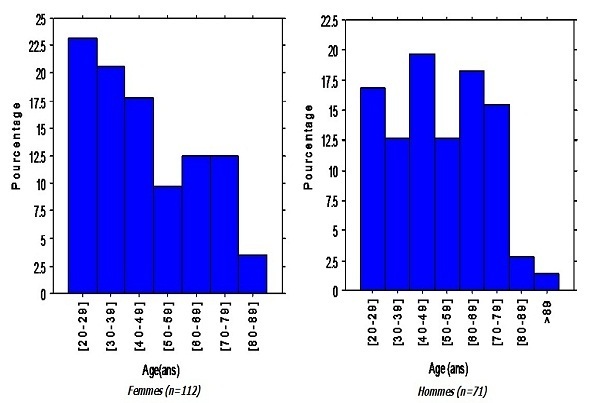
Répartition des patients selon l’âge par sexe

D'après le pourcentage élevé des femmes atteintes entre 20 et 39 ans et celui en régression entre 40 et 89 ans, on remarque que les femmes sont plus exposées à ce type de pathologies, durant la période reproductive, par contre elle est généralement plus fréquente chez l'homme, après la quarantaine (Figure 1). 146 cas (79.7%) de TVP isolée ont été recensés, tandis que 37 autres (20.2%), présentaient une EP, dont 16 parmi eux, avaient une TVP associée (Tableau 1).

**Figure 2:**
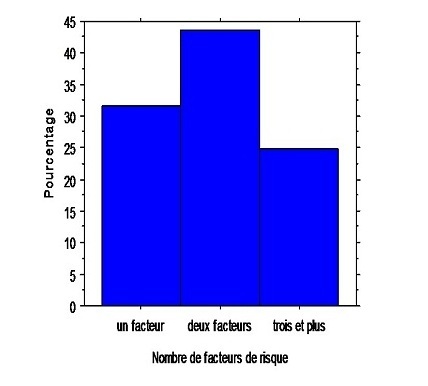
Répartition des cas selon le nombre de facteurs de risque

Dans le cas des patients atteints d'une TVP, les facteurs de risque à fort potentiel thrombogène, sont surtout: l'immobilité, l'hypertension, la chirurgie et la contraception orale. Pour les malades atteints d'EP, les facteurs de risque particulièrement incriminés sont: l'immobilité, la chirurgie, l'hypertension et les fractures (Tableau 2). L'antécédent personnel était présent dans 12 .02% des cas (Tableau 2). Plusieurs facteurs de risque ont été notés chez 24.7% des patients, 43.5% en avaient deux et 31.6% des patients, n'en présentaient qu'un seul (Figure 2), alors que 16 cas (8.7%) dont (14 TVP et 2 EP) n'avaient pas de facteurs de risque déterminés.

**Tableau 2:** Répartition des patients selon les facteurs favorisants

Facteurs de risque	Patients avec TVP (n = 146)	Patients avec EP (n = 37)
Age (années)	48±18.1	50 ±17.7
Tabagisme	11(7.5)	3(8.1)
Obésité	4(2.7)	5(13.5)
Immobilité	39(26.7)	14 (37.8)
Chirurgie	18(12.3)	10(27)
Fractures	11(7.5)	8(21.6)
Grossesse	4(2.7)	0(0)
Post-partum	15(10.2)	5(13.5)
Post abortum	2(1.3)	0(0)
Contraception orale	16(10.9)	2(5.4)
Cancer	10(6.8)	4(10.8)
Diabète	9(6.1)	2(5.4)
Hypertension	21(14.3)	9(24.3)
Maladie de Behcet	4(2.7)	0(0)
Syndrome néphrotique	9(6.1)	0(0)
Insuffisance cardiaque	8(5.4)	0(0)
AVC	2(1.3)	2(5.4)
Infarctus du myocarde	1(0.6)	3(8.1)
Varices	7(4.7)	1(2.7)
Tuberculose pulmonaire	6(4.1)	0(0)
Antécédents personnels de MTEV	15(10.2)	7(18.9)
Indeterminé	14(9.5)	2(5.4)

Valeurs exprimées en n(%), moyenne ± écart type pour l’âge; AVC : accident vasculaire cérébral; METV: Maladie thromboembolique veineuse; EP: embolie pulmonaire

Sur les 158 malades atteints de TVP des membres inférieurs, la TVP proximale était de 97.5% des cas et la TVP distale de 2.5% des cas, avec une prédominance de la localisation gauche dans 101 cas, suivie de la localisation droite dans 47 cas et de 10 cas pour la localisation bilatérale (Tableau 3). En revanche, seulement 4 cas de TVP des membres supérieurs avec une prédominance de la localisation gauche, ont été recensés (Tableau 3), dont deux femmes atteintes respectivement de cancer du sein et de lymphome malin et deux hommes atteints, ne présentant pas de cause apparente.

**Tableau 3:** Répartition des cas de TVP selon la localisation

Membre inférieur (n = 158)	TVP gauche (n = 101)	TVP droite (n = 47)	TVP bilatérale (n = 10)
Iliofémorale + VCI	0(0)	3(6.3)	0 (0)
Iliofémorale	17 (16.8)	5 (10.6)	2 (20)
Ilio-fémoro-poplité	34 (33.6)	10 (21.2)	3 (30)
Fémoropoplité	37 (36.6)	18(38.2)	5(50)
Fémorale	2 (1.9)	8 (17)	0(0)
Poplité	8 (7.9)	2 (4.2)	0(0)
Tibiale	3 (2.9)	1(2 .1)	0(0)
**Membre supérieur (n =4)**	**TVP gauche (n = 3)**	**TVP droite (n = 1)**	**TVP bilatérale (n = 0)**
Axillo- sous clavière et humérale	3(100)	0 (0)	0(0)
Axillo- sous clavière	0(0)	1 (100)	0(0)

* Valeurs exprimées en n(%); VCI : veine cave inférieure; TVP : Thrombose veineuse profonde

## Discussion

L'incidence des événements thromboemboliques veineux, augmente avec l’âge qui est un facteur de risque thromboembolique indépendant. Ce risque est d'autant plus important qu'il ya avec l’âge, une incidence accrue de comorbidités associées (interventions chirurgicales, immobilité, ou cancer), favorisant le développement des thromboses veineuses [5].

L'incidence d'un premier épisode thromboembolique veineux, passe d'un taux négligeable avant 15 ans [3]. Selon l’étude de Oger et al, l'incidence de la MTEV augmente nettement avec l’âge et atteint 1% chez les patients de plus de 75 ans. Cette incidence est 2 fois plus élevée que celle du groupe d’âge compris entre 60 et 74 ans [6]. Les femmes en âge de procréer sont plus touchées que les hommes dans la même tranche d’âge. Cette différence, est dûe à l'association de l’événement thromboembolique, à la grossesse et à l'utilisation de la contraception orale. En revanche, le risque chez les femmes âgées est substantiellement inférieur à celui des hommes dans le même groupe d’âge [1, 3, 6].

Les modifications physiologiques qui s'opèrent chez le sujet âgé, constituent avec d'autres facteurs de risque, un terrain favorable à l'installation d'une MTEV. La diminution significative du diamètre et de la vélocité au repos de la veine fémorale commune au- delà de 60 ans, est un facteur prédisposant aux TVP dû à une réduction significative du flux dans cette veine fémorale commune [7]. L'alitement et la réduction de la mobilité, favorisent la dilatation veineuse et la stase sanguine [7].

Une réduction du retour veineux causée par la diminution de la fonction pompe des muscles du mollet avec l’âge, a été soulignée par une étude pléthysmographique menée sur des sujets de 23 à 40 ans et d'autres de 60 à 83 ans [7]. Par ailleurs, une augmentation des taux des facteurs de coagulation VIII, V, VII, IX et une variation des marqueurs prothrombotiques étaient corrélées à l’âge [7].

Selon les résultats de notre étude, il en ressort que certains facteurs thrombogènes étaient plus fréquents que d'autres, à savoir: l'immobilité, l'hypertension, la chirurgie et la contraception orale, en cas de TVP et l'immobilité, la chirurgie, l'hypertension et les fractures en cas d'EP.

Fondée sur des arguments physiopathologiques, l'immobilisation a été considérée comme étant un facteur de risque de thromboembolie veineuse. Ainsi, la position allongée, peut conduire à un dysfonctionnement musculaire et diaphragmatique, ce qui diminue le flux veineux dans les jambes et provoque la stase veineuse. Cette stase, peut à son tour induire un état d'hypercoagubilité en activant la voie extrinsèque de la coagulation par l'intermédiaire d'une hypoxémie, par la production de lésions endothéliales ou par la réduction de l'activité fibrinolytique [8].

Plusieurs études ont conclu que l'immobilisation était associée au risque de la MTEV: Isma N et al [9], ont trouvé que pour 17% des patients atteints de TVP et 18% atteints d'EP, le facteur de risque était l'immobilisation. Selon Ouldzein H et al [10], l'alitement représente 35% des patients atteints d'EP. Une autre étude de Healy. B et al [11], a montré que l'immobilité due à une position assise prolongée au travail, était associée à un risque de MTEV 2.8 fois plus élevé. Pour 36.3% des femmes, la MTEV était associée à l'immobilisation, selon une étude menée par Fletcher HM et al [12].

Une association positive entre la pression artérielle et la MTEV a été constatée dans l’étude de Goldhaber SZ et al qui a trouvé que l'hypertension est un prédicteur indépendant de l'EP chez les femmes [13]. Cependant, selon l’étude de Tsai et al [14], l'hypertension n’était pas un facteur de risque de la MTEV.

Quant à la chirurgie, elle augmente de 20 fois le risque de MTEV lié au type et à la durée du geste opératoire, à la pathologie sous-jacente ou au terrain du patient pouvant aggraver la stase veineuse, élément prépondérant de la thrombogénèse [15, 16]. Sans prophylaxie de routine, le taux de TVP chez les patients opérés en chirurgie générale, a été évalué de 10 à 40% [17]. En général, les chirurgies abdominales, pelviennes, orthopédiques, neurochirurgicales et oncologiques exposent le patient à un risque important de MTEV, favorisé par l'immobilisation, la sténose veineuse secondaire et des lésions endothéliales [18]. En effet, la chirurgie est associée à une coagulation active, à une diminution transitoire de la fibrinolyse. Une suractivation de la thrombine, ainsi que des taux élevés de l'inhibiteur de l'activateur du plasminogène 1(PAI-1) au cours de la période périopératoire, ont été décrits [18].

Par ailleurs, la prise de contraceptifs œstroprogestatifs, doit être systématiquement recherchée chez les patientes en âge de procréer. En effet, la contraception œstroprogestative orale a longtemps été associée à un risque thrombotique veineux accru, globalement multiplié par quatre [19–22]. Ce risque varie selon la dose et le type du contraceptif utilisé. Ainsi, il est plus important, proportionnellement aux doses quotidiennes et plus élevé avec les progestatifs de 3^ème^ génération, par comparaison à ceux de 2^ème^ génération [19, 22–25]. Le risque lié à la contraception hormonale est modulé significativement avec l’âge, en particulier au-delà de 40ans, mais aussi en présence d'autres facteurs de risque [20]. Des anomalies de l'hémostase, telles que: la diminution de la protéine S et de l'antithrombine et la résistance acquise à la protéine C activée, observées sous contraception œstroprogestative, confèrent un terrain biologique favorable à la survenue des thromboses veineuses [26–31]. D'autres part, les taux de la SHBG (sexe hormone binding globulin) plus élevé avec les progestatifs de 3^ème^ génération qu'avec ceux de 2^ème^ génération, sont considérés comme un marqueur de risque de thrombose [32–36]. Les événements thromboemboliques sont fréquents après un traumatisme, notamment en cas de fractures. Ce risque est augmenté de 13 fois dans le cas d'un traumatisme récent [37].

Quant à la TVP des membres supérieurs, elle constitue une localisation peu fréquente de la maladie thrombo-embolique et représentait 3 à 5% de l'ensemble des TVP mais actuellement, sa fréquence augmente notamment en raison de l'utilisation croissante des cathéters veineux centraux (chimiothérapie, alimentation parentérale, hémodialyse), [38].

Le risque de la MTEV, augmente proportionnellement avec le nombre de facteurs prédisposants, 96% des patients présentaient au moins un facteur de risque reconnu d'après l’étude de Anderson FA et al [39]. D'autre part, ce risque est nettement plus important chez les patients ayant déjà présenté un événement veineux thromboembolique, et le risque cumulé de récidive après un premier épisode est très important, ce qui justifie de considérer la MTEV comme une pathologie chronique. Le risque de récidive est évalué de 5 à 10% par an [40–42]. Hansson retrouve un taux de récidive de 7% après un an, 21.1% après 5 ans de suivi en cas de premier épisode, alors que les récidives à 5ans sont de 27.9% après un 2^ème^ épisode [43]. Le risque de récidive est plus important en cas de thrombose proximale [43].

## Conclusion

En dépit de sa fréquence non alarmante, la MTEV est un ennemi redoutable sournois, pouvant aboutir à une morbi-mortalité accrue, qu'il est impératif de cerner en instaurant des mesures prophylactiques rigoureuses, adaptées au niveau du risque thrombotique. En dehors des méthodes physiques et des règles d'hygiène veineuse simples, qui doivent être appliquées à toutes les situations à risque, pour lutter contre la stase et accélérer le retour veineux, des traitements antithrombotiques sont prescrits en cas de risque modéré ou élevé. D'autres part, en médecine préventive, des mesures hygiénodiététiques s'imposent en priorité, renforçant ainsi les moyens thérapeutiques mis en œuvre, dans la prise en charge du syndrome métabolique.
